# Deconvoluting Post-Transplant Immunity: Cell Subset-Specific Mapping Reveals Pathways for Activation and Expansion of Memory T, Monocytes and B Cells

**DOI:** 10.1371/journal.pone.0013358

**Published:** 2010-10-14

**Authors:** Yevgeniy A. Grigoryev, Sunil M. Kurian, Zafi Avnur, Dominic Borie, Jun Deng, Daniel Campbell, Joanna Sung, Tania Nikolcheva, Anthony Quinn, Howard Schulman, Stanford L. Peng, Randolph Schaffer, Jonathan Fisher, Tony Mondala, Steven Head, Stuart M. Flechner, Aaron B. Kantor, Christopher Marsh, Daniel R. Salomon

**Affiliations:** 1 Molecular and Experimental Medicine, The Scripps Research Institute, La Jolla, California, United States of America; 2 Biomarker Discovery Sciences, Pharmaceutical Product Development (PPD), Menlo Park, California, United States of America; 3 Scripps Center for Organ and Cell Transplantation, La Jolla, California, United States of America; 4 Roche, Palo Alto, California, United States of America; 5 The Scripps Research Institute (TSRI) DNA Microarray Core Facility, La Jolla, California, United States of America; 6 Glickman Urological Institute, The Cleveland Clinic, Cleveland, Ohio, United States of America; New York University, United States of America

## Abstract

A major challenge for the field of transplantation is the lack of understanding of genomic and molecular drivers of early post-transplant immunity. The early immune response creates a complex milieu that determines the course of ensuing immune events and the ultimate outcome of the transplant. The objective of the current study was to mechanistically deconvolute the early immune response by purifying and profiling the constituent cell subsets of the peripheral blood. We employed genome-wide profiling of whole blood and purified CD4, CD8, B cells and monocytes in tandem with high-throughput laser-scanning cytometry in 10 kidney transplants sampled serially pre-transplant, 1, 2, 4, 8 and 12 weeks. Cytometry confirmed early cell subset depletion by antibody induction and immunosuppression. Multiple markers revealed the activation and proliferative expansion of CD45RO^+^CD62L^−^ effector memory CD4/CD8 T cells as well as progressive activation of monocytes and B cells. Next, we mechanistically deconvoluted early post-transplant immunity by serial monitoring of whole blood using DNA microarrays. Parallel analysis of cell subset-specific gene expression revealed a unique spectrum of time-dependent changes and functional pathways. Gene expression profiling results were validated with 157 different probesets matching all 65 antigens detected by cytometry. Thus, serial blood cell monitoring reflects the profound changes in blood cell composition and immune activation early post-transplant. Each cell subset reveals distinct pathways and functional programs. These changes illuminate a complex, early phase of immunity and inflammation that includes activation and proliferative expansion of the memory effector and regulatory cells that may determine the phenotype and outcome of the kidney transplant.

## Introduction

A major challenge for the field of transplantation is the lack of understanding of genomic and molecular drivers of early post-transplant immunity. The early inflammatory response is initiated by ischemia/reperfusion, activation of innate immunity and subsequent alloantigen-primed T cell recruitment, activation and proliferative expansion [1]–[5]. The early immune response creates a complex milieu that contributes significantly to the course of ensuing events and the ultimate outcome of the transplant including acute and chronic rejection [6]–[9]. Thus, profiling the mechanisms of early immunity is essential.

The last several decades of evolving clinical practice in kidney transplantation has focused on increasing graft survival by reducing the risk of acute rejection, while enhancing the safety profiles of the drug regimens employed [10], [11]. It is now common to use induction therapy with anti-lymphocyte antibody preparations to profoundly deplete the cellular immune system immediately at the time of transplantation [12]–[14]. Induction in combination with current drug therapies reduces acute rejection incidence to less than 15% in the first year [15], [16]. Unfortunately, these dramatic results in the short term reduction of acute rejection have not directly translated to long term immune tolerance with successful drug withdrawal or even a significant reduction in the incidence of chronic rejection or chronic allograft nephropathy with interstitial fibrosis and tubular atrophy (CAN/IFTA) [17]–[19]. Moreover, several studies in which biopsies were performed by protocol rather than by clinical indication between 6 and 12 months after transplantation revealed that up to 15% of patients demonstrated evidence of an active immune/inflammatory response despite no evidence of transplant dysfunction [17], [20]. Thus, there is a pressing medical need to understand what changes evolve in this early post-transplant period that allows the immune response to reemerge intact and target the transplant for immune-mediated rejection and injury.

The objective of the current study was to mechanistically deconvolute the early immune response in kidney transplant patients after antibody induction therapy by purifying and profiling the constituent peripheral blood cell subsets using two complementary technologies. First, we employed the novel SurroScan™ laser scanning cytometry [21], [22], [23] technology on whole blood cell populations to create a comprehensive survey of well-established cell surface marker expression from 10 consecutively enrolled transplant patients and 5 healthy controls. The transplant patients were serially sampled from pre-transplant (Pre-TX) to 12 weeks post-transplant (Post-TX). Second, genome-wide differential gene expression profiling was done. We purified and analyzed CD4^+^ and CD8^+^ T lymphocytes, CD14^+^ monocytes, and CD19^+^ B cells. Whole blood and subset-specific gene expression profiles were used to populate and map molecular pathways as a function of time and population.

This novel approach to cell subset-based deconvolution revealed the profound changes in blood cell composition and the activation of multiple molecular pathways that occurs early Post-TX. Moreover, each immune cell subset revealed a distinct set of pathways and functional programs. Indeed, these subset-specific changes illuminate a complex, early phase of alloimmunity characterized by the activation and proliferative expansion of the memory effector and regulatory T cells that play major roles in determining the outcome of the kidney transplant. We also demonstrated significant activation and proliferative expansion of CD14 monocytes that are linked to ischemia reperfusion injury, innate immunity and the transition to adaptive immunity. Finally, we demonstrated the progressive activation of B cells by 12 weeks consistent with early recruitment of the humoral immune system post-TX.

## Results

### Patient population

**Table 1** presents the clinical characteristics collected from 10 kidney transplant patients. Five patients were converted to sirolimus immunosuppression at 12 weeks, two patients experienced acute rejection episodes at 7 and 10 months, and creatinine levels out to 12 months are provided. All patients were enrolled in a single, consecutive series after consent. **Table 2** lists all the samples obtained for this study.

**Table 1:** Transplant Patient Characteristics.

Recipient characteristics	Pt. 1	Pt. 2	Pt. 3	Pt. 4	Pt. 5	Pt. 6	Pt. 7	Pt. 8	Pt. 9	Pt. 10
**Age**	67	41	44	29	65	58	41	67	44	60
**Sex**	M	M	M	F	M	M	M	M	F	F
**Race/Ethnicity**	Black	White	Filipino	White	White	Hispanic	Asian	Hispanic	Black	White
**Type of renal disease^ 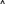 ^**	HTN	HTN	HTN	HUS	Type 2 DM	HTN & MPGN	IgA	UNK	HTN & ?GN	Type 1 DM
**Thymoglobulin Induction**	Yes	Yes	Yes	Yes	Yes	Yes	Yes	Yes	Yes	Yes
**Donor** #	DD	DD	DD	Living	DD	DD	Living	DD	Living	Living
**Conversion** *	No	Sirolim-us	Sirolim-us	No	No	Sirolim-us	Sirolim-us	No	Sirolim-us	No
**MPA dose/level at 12 weeks** †	2gm/0.8	1.25gm/5.0	2gm/2.2	2gm/4.2	2gm/2.9	1.5gm/3.0	3gm/2.4		3gm/3.3	2gm/1.2
**Rejection (Yes/No)**	Yes	Yes	No	No	No	No	No	No	No	No
**Type of Rejection**	Banff 1b‡	Banff 1a	None	None	None	None	None	None	None	None
**Rejection months Post-Tx**	10	7	None	None	None	None	None	None	None	None
**Creatinine - Month 1**	1.1	2	2	0.9	1.6	3.9	1.6	1.9	1.4	1.1
**Creatinine - Month 3**	0.9	1.7	1.8	0.9	1.2	1.9	1.2	NA	1.0	1.2
**Creatinine – Month 12**	1.8	3.0	1.8	0.8	1.7	1.6	1.2	1.5	1.0	1.3

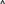
 HUS  =  Hemolytic Uremic Syndrome; Type 2 DM  =  Type 2 Diabetes Mellitus; IgA  =  IgA nephropathy; HTN  =  hypertension; MPGN  =  membranoproliferative GN; ?GN  =  glomerulonephritis of ? etiology; Type 1 DM  =  Type 1 Diabetes Mellitus;

† MPA  =  Mycophenolic acid.

# DD  =  Deceased donor.

‡ Banff  =  international classification schema for kidney transplant pathology. Banff 1a and 1b are different forms of acute tubulointerstitial cellular acute rejection.

*All patients were started on FK506/CellCept with no Prednisone. Conversions from FK506 to sirolimus were done at approximately 3 months post-transplant as tolerated.

**Table 2:** Summary of samples for whole blood and purified cell subset analysis.

Samples	Whole Blood	CD4	CD8	CD14	CD19
Time					
**Pre-Tx**	8	5	4	2	1
**Week1**	9	1	0	2	0
**Week2**	9	7	7	10	5
**Week4**	10	2	0	2	0
**Week8**	7	2	1	2	0
**Week12**	9	9	7	10	4
**Healthy Normal Controls**	5	5	5	5	5

### Cytometry results demonstrate the selective activation and proliferative expansion of CD45RO^+^CD62L^−^ effector memory CD4 T cells

A primary objective was to determine differences in immune/inflammatory cell populations in the early Post-TX period. SurroScan™ microvolume laser scanning cytometry provides quantitative measurements of absolute cell counts per microliter of blood and simultaneous measurement of hundreds of cell surface markers defining multiple cell populations [22]. Of 1083 variables measured at each time point, 441 were significantly different at week 1 and 259 at week 12 (p<0.01) (**Table 3**).

**Table 3:** Summary of the number of significant variables as a function of time defined by cytometry from a total of 1083 variables.

		Pre-Tx vs. Wk1	Pre-Tx vs. Wk2	Pre-Tx vs. Wk4	Pre-Tx vs. Wk8	Pre-Tx vs. Wk12	Wk1 vs. Wk2	Wk1 vs. Wk4	Wk1 vs. Wk8	Wk1 vs. Wk12	Wk2 vs. Wk4	Wk2 vs. Wk8	Wk2 vs. Wk12	Wk4 vs. Wk8	Wk4 vs. Wk12	Wk8 vs. Wk12
**# Subjects**		10	9	10	8	10	8	9	8	9	9	8	9	8	10	8
**P-value**	**-log (p values)** *															
**P<0.0001**	>4	96	91	81	9	53	3	16	25	24	7	9	29	0	0	1
**P<0.001**	>3	202	222	162	84	118	27	74	98	82	35	60	82	5	14	4
**P<0.01**	>2	441	423	374	256	259	153	294	294	349	133	254	290	43	78	42
**P<0.05**	>1.3	565	585	500	408	416	302	421	446	514	293	403	465	174	254	130

*Cytometry data shown in this manuscript are reported as –log (p values). They can be converted to traditional p values as shown here.

**Figure 1:**
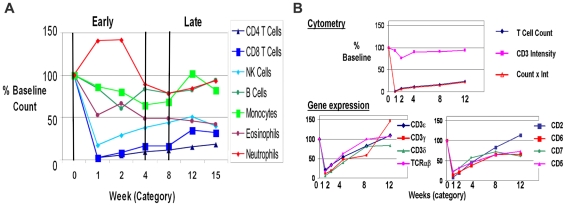
Serial cytometry profiling of cell subset populations shows distinct patterns of depletion and recovery early post transplantation. (**A**). Absolute cell counts were determined and expressed as a percent of the counts pre-treatment. Multiple changes were observed in peripheral blood cell populations following transplantation, induction antibody therapy and drug-based immunosuppression. The different major cell populations show distinct patterns of depletion, gain and recovery as a function of time in the first 12 weeks, which we have defined as the “early” post transplant period. The gradual recovery of each of the subsets in time after transplant demonstrate that a proliferative expansion of at least subpopulations of these cells is underway despite the many individual differences observed. (**B**). T cell counts and mRNA show the same pattern of depletion and recovery. Cytometry variables for comparison to gene expression included: cell counts and expression intensity – mean per cell. CD3 intensity per cell is constant with treatment. The product of count and intensity (Count x Int) integrates the expression of each marker on each subpopulation at each time point and is the sum of the mean intensities of the given marker multiplied by all the cells identified in that population at any given time point. Count x Int. gives the same relative to baseline as absolute counts, indicating that the loss of T cells, not CD3 intensity, is the key changing parameter. Gene expression shows a consistent pattern for the 8 variables representing known T cell markers and correlates well with T cell counts. This is the first example of a proteogenomic validation of the results.

Thymoglobulin induction also causes a rapid and profound reduction in multiple cell populations [24]–[26]. The changes in absolute cell counts for each major cell population are given in **Figure 1A**. Consistent with a previous report following longer term cell reconstitution [13], T cell counts returned to only 20% of Pre-TX levels at 12 weeks and CD4 levels lagged behind CD8. The excellent correlations of T cell markers with the gene expression data we will describe later demonstrate the first proof for integrating cytometry and DNA microarray profiling (**Figure 1B**).

The distribution of different CD4 T cell subsets evolved from Pre-TX to 12 weeks (**Table 4**). Naïve and memory CD4 subsets differ by expression of CD62L and either CD45RO (memory) or CD45RA (naïve). At 12 weeks, there was a significant expansion of memory effector CD4 cells (CD45RA^−^CD62L) from 30% Pre-TX to 45% at 12 weeks (p<0.01). These memory effector CD4 also significantly increased their expression of multiple activation markers: CD60 [27], CCR5 (CD195) [28], CD69 [29], CD86 [30], CD132 [31], CD27 [32] and HLA DR [33]. While the CD4^+^CD25^+^ T cells are also depleted by induction, they increased significantly (2.5-fold) by 12 weeks. Reciprocally, the naïve CD4 population was significantly lower at 12 weeks, going from 28% to 12% of the total CD4 T cells (p<0.001). In contrast, there were no significant changes in central memory or terminal effector CD4 numbers.

**Table 4:** Cytometry results defining CD4 T and CD8 T cell subsets.

Subtype	Population^ 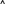 ^	-log(p)#	Effect Size†	Mean Ratios	Mean Wk 0*	Mean Wk 12*
CD4	CD45RA^−^ CD62L^−^ (effector memory)	2.46	1.29	1.44	0.30	0.45
CD4	CD45RA^−^ CD62L^+^ (central memory)	0.78	0.28	1.10	0.34	0.37
CD4	CD45RA^+^ CD62L^+^ (naïve)	3.49	0.96	0.44	0.28	0.12
CD4	CD45RA^+^ CD62L^−^ (terminal effector)	0.35	0.35	0.78	0.08	0.06
CD4	CD45RO^−^ CD62L^+^ (naïve)	2.36	0.99	0.40	0.21	0.09
CD4	CD45RA^+^ CD28^+^	4.13	1.22	0.44	0.38	0.16
CD4	CD45RA^−^ CD28^+^	1.32	0.77	1.24	0.53	0.67
CD4	CD60^+^ CCR5^−^	1.90	1.28	1.68	0.19	0.28
CD4	CD60^+^ CCR5^+^	2.41	1.24	2.47	0.06	0.15
CD4	CD60^−^ CCR5^−^ (unactivated)	3.66	2.63	0.57	0.62	0.36
CD4	CD60^−^ CCR5^+^	2.41	1.36	1.76	0.08	0.16
CD4	CD132^+^	2.71	1.33	3.31	0.05	0.13
CD4	CD183^+^	2.13	1.24	1.77	0.18	0.31
CD4	CD197^+^	2.01	1.12	0.58	0.47	0.27
CD4	CD25^−^	2.44	1.47	0.90	0.93	0.84
CD4	CD25^+^ (includes putative T regs)	2.41	1.45	2.49	0.07	0.16
CD4	CD45RB^+^ CD27^+^	3.63	1.41	0.49	0.50	0.24
CD4	CD49d^+^	2.49	1.25	1.14	0.78	0.87
CD4	CD69^+^	1.77	1.23	2.56	0.03	0.06
CD4	CD86^+^	2.07	1.55	5.38	0.03	0.07
CD4	HLA DR^+^	1.62	1.13	2.94	0.09	0.21
CD4	IL-15Ra^+^	2.72	1.28	2.38	0.03	0.08
CD8	CD45RA^−^ CD62L^−^ (effector memory)	0.87	0.43	1.22	0.25	0.30
CD8	CD45RA^−^ CD62L^+^ (central memory)	0.68	0.41	1.21	0.10	0.13
CD8	CD45RA^+^ CD62L^+^ (naïve)	0.15	0.09	1.05	0.30	0.34
CD8	CD45RA^+^ CD62L^−^ (terminal effector)	1.59	0.90	0.70	0.35	0.23
CD8	CD25^−^	0.45	0.38	1.01	0.96	0.98
CD8	CD25^+^	0.45	0.38	0.73	0.04	0.02
CD8	CD60^−^ CCR5^−^ (unactivated)	3.59	1.17	0.70	0.53	0.38
CD8	CD60^+^ CCR5^−^	2.41	0.96	2.81	0.06	0.09
CD8	CD60^+^ CCR5^+^	0.98	0.83	1.55	0.09	0.11
CD8	CD57^+^	1.99	NA	0.52	0.14	0.06
CD8	CD27^+^	1.93	NA	1.18	0.66	0.78
CD8	CD38^+^	1.60	NA	2.15	0.18	0.35

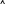
Nomenclature of cell populations are based on the antigen expression: +  =  positive, −  =  negative.

# Significant using the term “–log(p)” equals 1.30 or greater. In standard p values that is equivalent to p≤0.05 (see Table 2).

† Effect size  =  Mean difference between groups divided by the weighted standard deviation (SD) is presented as an absolute value. This is another metric for significance such that greater than 0.80 equals significant expression difference.

*CD4 T cell subsets are given as a fraction of total CD4 cells. CD8 T cell subsets are given as a fraction of total CD8 cells.

By 12 weeks total CD8 cells were 40% of baseline (**Figure 1A**). Parallel to the CD4 data, there was an observed increase in activated memory effector CD8 cells (CD45RA^−^CD62L^−^; 25% to 30%; **Table 4**). While this did not reach statistical significance, the parallel increase in CD8^+^CD45RA^−^ cells from 35% to 42% was significant (p<0.05). Multiple activation markers were also upregulated on CD8 including CD60, CCR5, CD27, while CD25 and CD57-expressing subsets were decreased by 25–50% at 12 weeks. In contrast to CD4, there was a significant decrease in terminal effector CD8 T cells from 35% to 23% (p<0.05).

**Figure 2:**
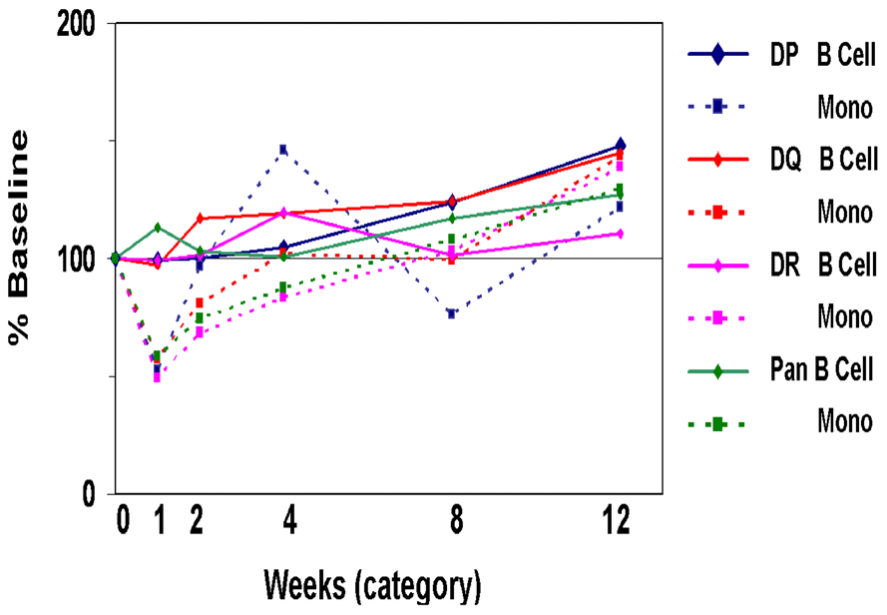
HLA Class II intensity levels for B cells and monocytes increase in time. HLA Class II expression intensity as percent of baseline is shown on the Y-axis, while weeks post transplant/induction is shown on the X-axis. Plotted are intensities for antibodies staining HLA-DP, DQ, DR and a pan HLA-reactive antibody for B cells and monocytes. These results clearly show the progressive upregulation of HLA antigen expression on both cell types consistent with a progressive immune activation that is supported by concomitant expression of multiple activation markers as described in the Results.

B cells and monocyte numbers dipped between weeks 1 and 4 but recovered to near baseline by 12 weeks (**Figure 1A**). Cell counts were well correlated to gene expression for B cell markers (CD19-21 and CD72; data not shown), another benchmark for integrating cytometry and gene expression. B cells expressing the activation marker CD38 were significantly higher 12 weeks Post-TX going from 31% to 45% (p<0.05; **Table 5**), consistent with the role for this molecule in responses to T cell dependent protein antigens [34]. In addition, B cells also demonstrated a significant increase of cells expressing the activation markers CD5, CD40, CD95 between early and late (data not shown). The number of monocytes in whole blood was also significantly higher by week 12 (2.1–2.4 fold; **Table 5**) and the TLR2^+^ (CD282) population also increased. Additional evidence for B cell and monocyte activation post-transplant are the progressive increases in HLA expression in both peaking at week 12 (**Figure 2**).

**Table 5:** CD14 Monocytes and CD19 B cell subsets.

Subtype	Population^ 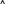 ^	-log(p)	Effect Size^†^	Mean Ratios	Mean Wk 0*	Mean Wk 12*
**B cells**	CD20^+^CD38^-^	1.52	0.94	0.80	0.69	0.55
**B cells**	CD20^+^CD38^+^	1.52	0.94	1.43	0.31	0.45
**B cells**	CD5^-^CD20^+^	0.71	0.28	1.01	0.96	0.97
**B cells**	CD5^+^CD20^+^	0.71	0.28	0.79	0.04	0.03
**Mono**	CD20^-^HLADR^-^	2.01	0.51	2.12	0.03	0.05
**Mono**	CD20^-^HLADR^+^	1.86	0.50	0.97	0.97	0.95
**Mono**	CD282^-^	2.30	1.06	2.41	0.01	0.02
**Mono**	CD282^+^	2.32	1.05	1.10	0.92	0.98

†
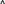
*See Table 4 for explanations.

### Whole blood gene expression profiling demonstrates over 2000 differentially downregulated genes mapped to multiple functional networks

**Figure 3:**
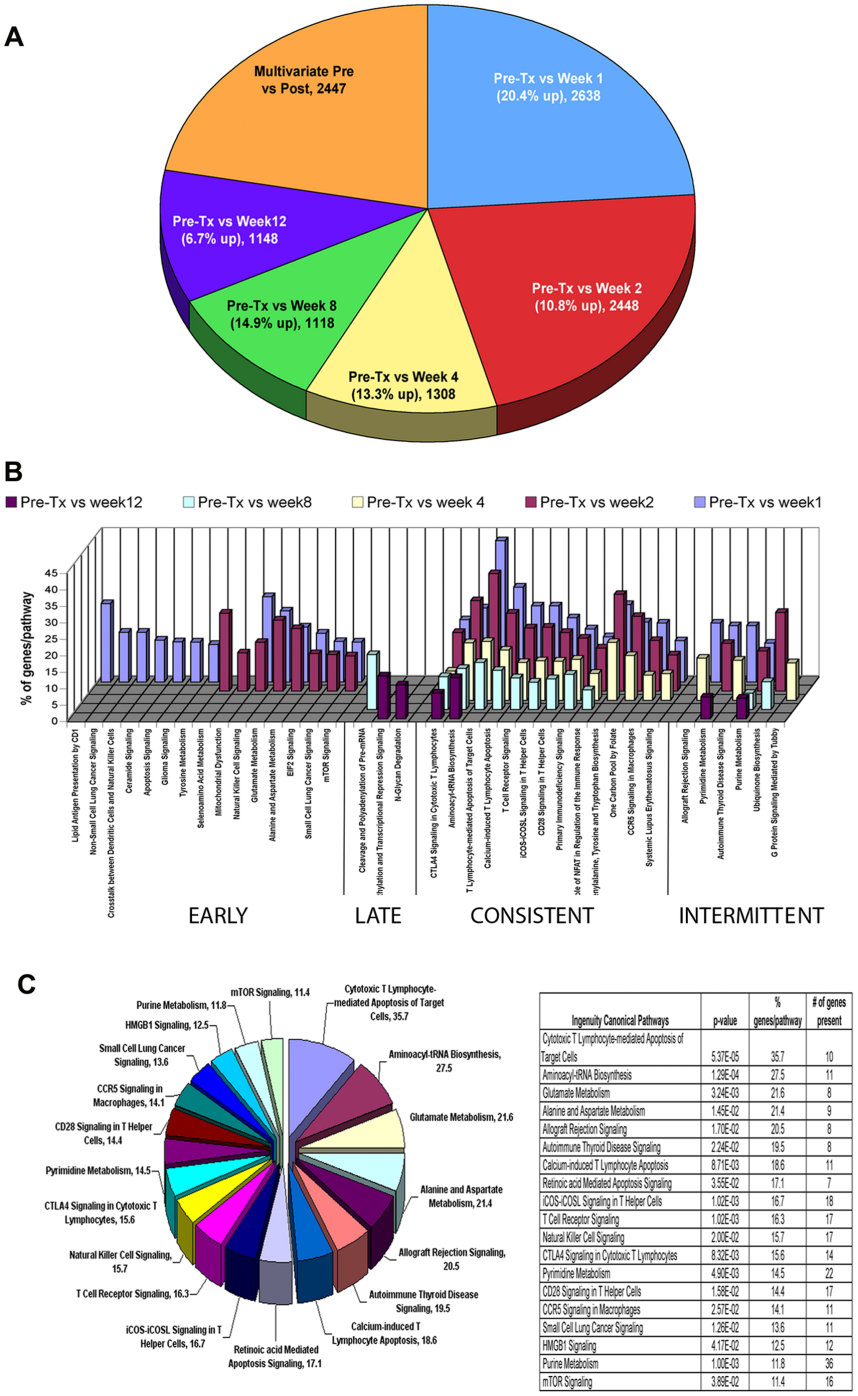
Gene expression profiling and functional analysis of whole peripheral blood. (**A**). A Pie chart summary for the number of differentially expressed genes in whole blood obtained in each time class-comparison analysis (p<0.001). The size of each slice represents the percentage of genes in that class based on the total of differentially expressed genes identified in all of the analyses done. Significant differential gene expression for each Post-TX timepoint was determined against the Pre-TX samples and these are represented in this figure as “Pre-TX vs. Week 1” and so on. In parallel, we performed ANOVA comparisons for all timepoints to determine what we have termed the “multivariate” genes that change significantly and differentially in a coordinate fashion at all timepoints post transplantation. (**B**). Functional analyses of the significant differentially expressed genes populating statistically significant Ingenuity pathways. The results for 5 timepoint comparisons: Pre-TX vs. week 1, 2, 4, 8 and 12 are shown in different colors. The Y-axis depicts the % of genes identified in our results vs. the total number of genes known to populate the pathway in the literature upon which Ingenuity mapping is based. We identified 4 different classes of differentially expressed genes mapping to these functional pathways: early, late, consistent and intermittent. The majority of genes are in the early and consistent classes. (**C**). Pie chart representing the 19 significant functional pathways populated by 134 significantly differentially expressed multivariate genes, with percentage of multivariate genes per total genes in a pathway. On the right, the 19 functional pathways shown in the pie chart are ranked by their p-value significance and includes the % genes populated per pathway, and the total number of genes identified for each pathway in our samples.

Differential gene expression for each Post-TX timepoint was determined. In a second analysis, ANOVA comparisons were done for all timepoints to determine genes that change in a coordinate fashion as a function of time (e.g. multivariate genes). These two analysis strategies are intended to be complementary. The number of differentially expressed genes obtained in each whole blood cell analysis is provided in **Figure 3A** and **Table 6**. The total number of differentially expressed and percent upregulated genes was highest at week 1 (2638; 20.4%) and decreased progressively by week 12 (1148; 6.7%). By ANOVA, we found 2447 multivariate genes, whose expression evolves in the continuum from Pre-TX to week 12. The majority of multivariate genes (90%) were also downregulated Post-TX (**Table S1**).

**Table 6:** Summary of differential gene expression from whole blood (WB) and the purified blood cell subsets.

Comparative analysis	Subset	Significant DE# genes (p<0.001)	Upregulated genes	% Upregulated
Pre-TX vs. Week 1	**WB** *	2638	539	20.4%
Pre-TX vs. Week 2	**WB** *	2448	265	10.8%
Pre-TX vs. Week 4	**WB** *	1308	174	13.3%
Pre-TX vs. Week 8	**WB** *	1118	167	14.9%
Pre-TX vs. Week 12	**WB** *	1148	77	6.7%
Multivariate: Pre vs. Post	**WB** *	2447	NA	NA
Pre-TX vs. Week 2	**CD4**	1584	726	45.8%
Pre-TX vs. Week 12	**CD4**	3358	1685	50.1%
Pre-TX vs. Week 2	**CD8**	574	295	51.3%
Pre-TX vs. Week 12	**CD8**	2030	1101	54.2%
Control vs. Week 2	**CD14**	312	251	80.4%
Control vs. Week 2 (Pre-TX normalized)	**CD14**	258	216	83.7%
Control vs. Week 12	**CD14**	616	301	48.8%
Control vs. Week 12 (Pre-TX normalized)	**CD14**	455	245	53.8%
Control vs. Week 2	**CD19**	208	121	58.1%
Control vs. Week 12	**CD19**	389	191	49.1%

*WB =  whole blood.

# DE  =  differentially expressed.

Next, we performed a functional analysis of gene expression comparing the Pre-TX gene expression to each of the serial time points sampled. We populated all possible functional pathways and then extracted only statistically significant pathways (p<0.05; **Methods S1**) for further analysis. Four classes of functional pathways were populated by this analysis: early (1–4 weeks), late (8–12 weeks), consistent (all timepoints) and intermittent (selected timepoints) (**Figure 3B**).

Most pathways were populated either early or consistently (see **Table S2** for gene details). Early pathways included NK Cell Signaling, CD1 Lipid Antigen Presentation and mTOR Signaling. Consistently populated pathways included CTLA4 Signaling in CTL, TCR Signaling, iCOS-iCOSL Signaling and CD28 Signaling. It is evident that these pathways include many of the known primary mechanisms of alloimmunity [35]–[38] and a key finding is that the majority of the genes populating these pathways are downregulated in the first 12 weeks coinciding with antibody induction and immunosuppression.

Using the 2447 multivariate genes by ANOVA, we populated 19 significant functional pathways with 134 significantly differentially expressed genes (**Figure 3C** **, Table S3**). These genes populate a combination of immune response and metabolic pathways. The top 5 pathways are CTL-mediated apoptosis (35.7% populated), aminoacyl-tRNA biosynthesis (27.5% populated), glutamate metabolism (21.6% populated), alanine and aspartate metabolism (21.4% populated), and allograft rejection signaling (20.5% populated).

### CD4 subset-specific gene expression reveals a unique spectrum of time-dependent upregulated genes mapping to multiple molecular networks

Two comparisons were done: Pre-TX vs. week 2 (early) and Pre-TX vs. week 12 (late). Pre-TX vs. week 2 revealed 1584 significantly differentially expressed genes. In sharp contrast to what was found by whole blood analysis, 45.8% of these CD4^+^ genes were upregulated (**Table 6**). Pre-TX vs. week 12 revealed 3358 genes differentially expressed of which 50.1% were upregulated. Thus, by week 12, the total number of differentially expressed and upregulated genes more than doubles in the CD4^+^ T cells.

**Figure 4:**
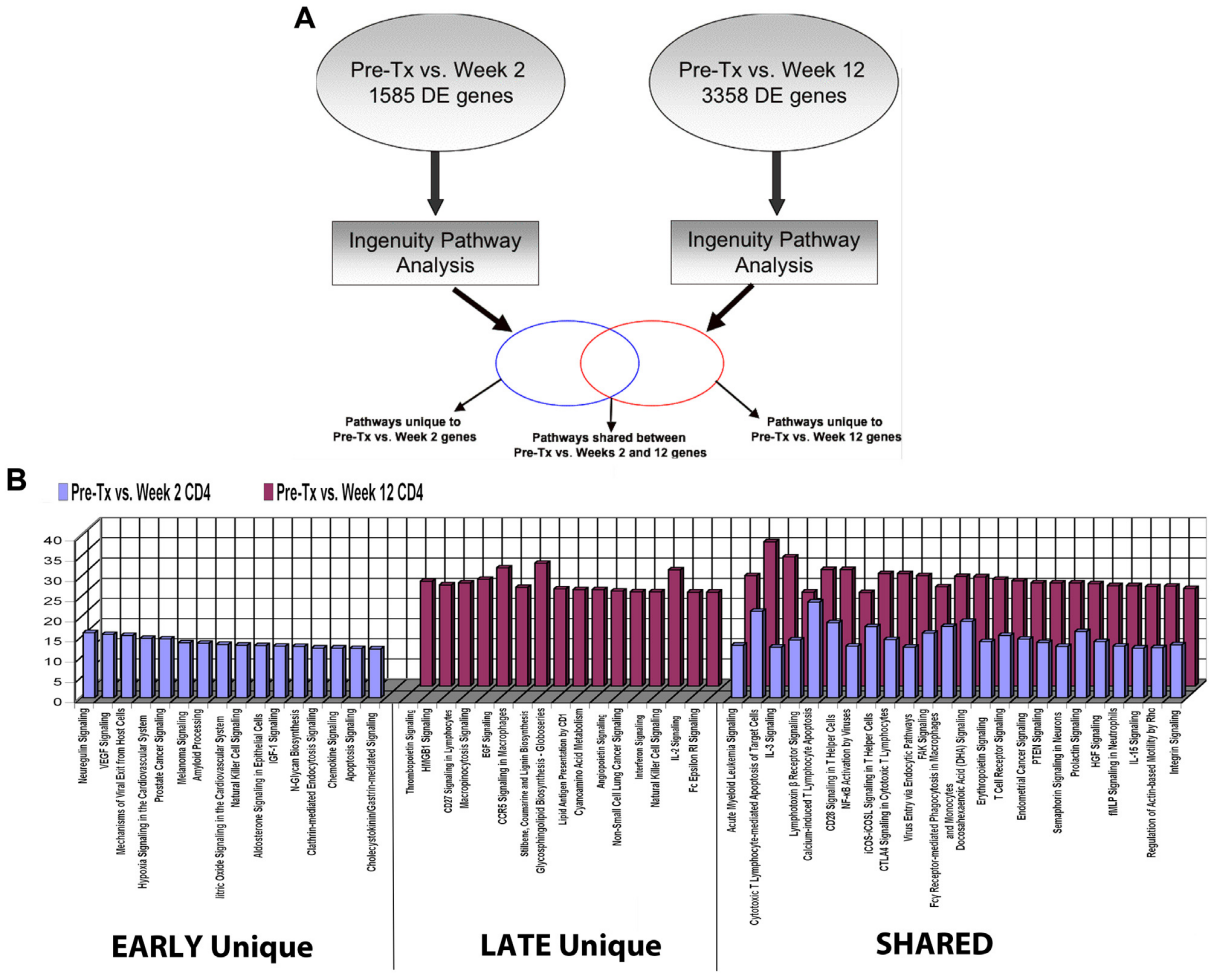
Gene expression profiling and functional analysis of the CD4 cell subset. (**A**). Schema representing our analysis of differential gene expression and the mapping of functional pathways for the CD4 T cell subset. This work was based on two time comparisons to clarify the evolution of changes during the first 12 weeks post transplantation: Pre-TX vs. Week 2 and Pre-TX vs. Week 12. (**B**). Functional pathways populated by genes differentially expressed early (blue), late (red) or shared at both timepoints Post-TX up to Week 12 in the CD4 T cell subset. The Y-axis represents % genes populated per pathway in our data based on the total number of genes identified by Ingenuity for each pathway.

Functional pathways were mapped to understand changes in differential gene expression between early and late Post-TX by the strategy shown in **Figure 4A**. It is evident that there are many unique pathways populated by genes expressed in either the early or late phase (**Figure 4B**). For example, VEGF, IGF1, chemokine, apoptosis and hypoxia signaling are unique early phase responses while CD28, angiopoietin, IL2, IL15 and Fce R1 signaling are unique in the late phase. Second, a large number of pathways are shared by both phases. However, it is important to note that these pathways are consistently populated by increasing numbers of differentially expressed genes in the late phase. These represent functional pathways triggered in the early phase that clearly are evolving with new gene members of the pathways being recruited and activated in the late phase of the immune response post transplant.

### Genes and pathways linked to CD8 activation are not common to those identified for CD4

Class comparisons of CD8 populations yielded 574 differentially expressed genes between Pre-TX and week 2 (51.3% upregulated) and 2030 genes between Pre-TX and week 12 (54.2% upregulated) (**Table 6**). Thus, the number of differentially expressed CD8^+^ genes almost quadruples by week 12.

**Figure 5:**
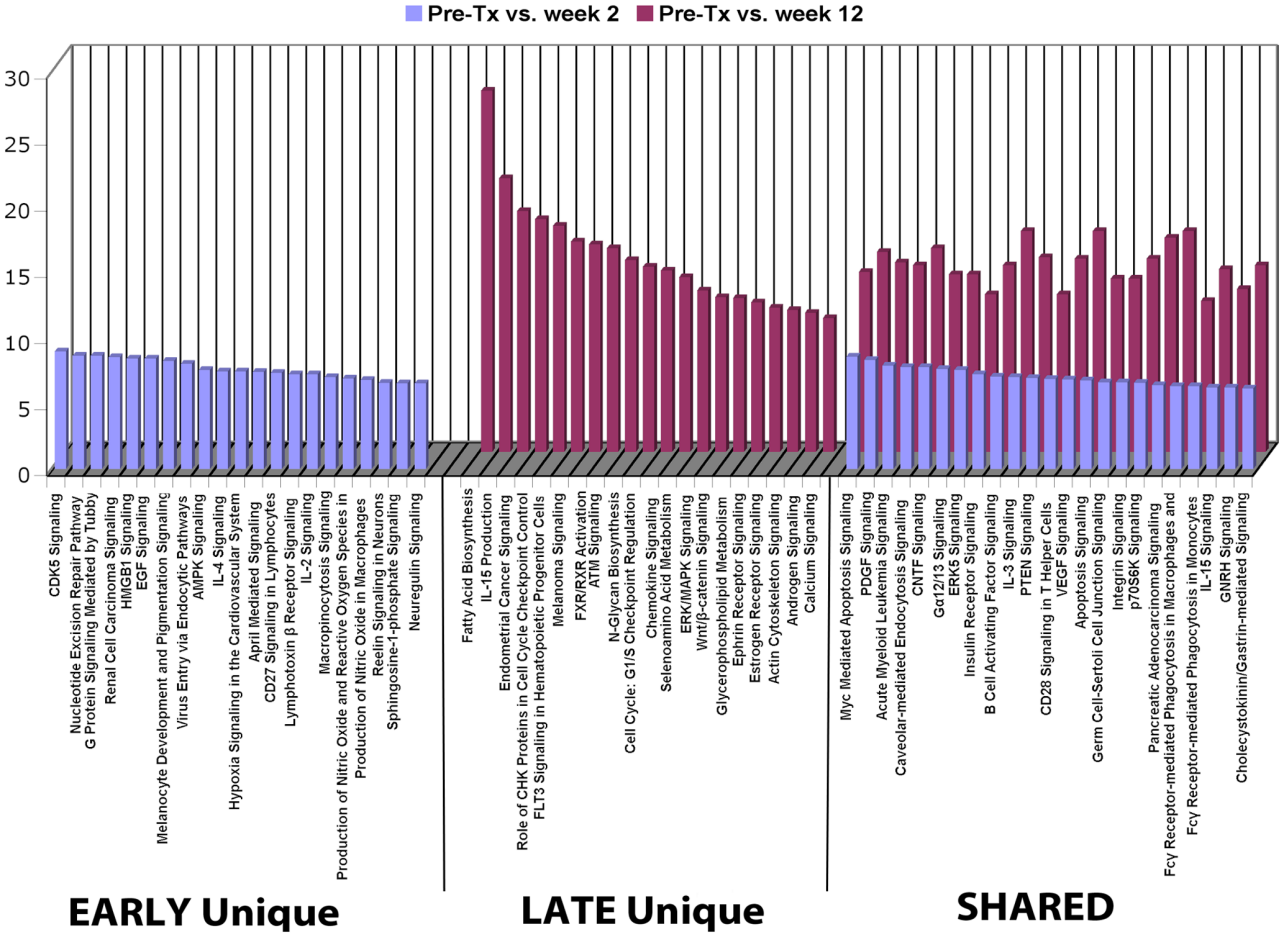
Gene expression profiling and functional analysis of the CD8 cell subset. Top 22 populated functional pathways for genes differentially expressed early (blue), late (red) or at both timepoints Post-TX in the CD8 T cell subset. Y-axis represents % genes present per pathway. For the complete list of functional pathways, see **Table S4**.

Comparison analysis identified pathways populated either early, late or at both phases Post-TX (see **Table S4** for entire list of pathways). The majority of pathways were either uniquely populated in the early phase (52 pathways) or expressed during both phases (46 shared pathways) compared to only 22 unique late pathways. The top 66 populated pathways are shown in **Figure 5**. Second, as seen with the CD4^+^ T cells, every shared pathway demonstrates a significant increase in the number of populating genes from week 2 to 12. Unique early pathways include IL2, IL4, sphingosine-1-phosphate and CD27 signaling. Unique late pathways include fatty acid biosynthesis, chemokine, Wnt-catenin, cell cycle signaling and IL15 production. Shared pathways increasingly populated in the late phase include IL3, CD28, integrin, VEGF and IL15 signaling, the latter matching the IL15 production pathway seen only in the late phase.

### CD14 monocytes reveal early activation of cytokine and inflammatory genes

Because of low cell yields, we substituted CD14-purified normal donor controls for the Pre-TX baselines and normalized the data by eliminating any genes differentially expressed between the controls and the two Pre-TX samples available. The class comparison at week 2 yielded 312 differentially expressed genes, 258 of which remained after normalization (84% upregulated; **Table 6**). At week 12, we identified 616 genes, 455 of which remained after normalization (54% upregulated).

**Figure 6:**
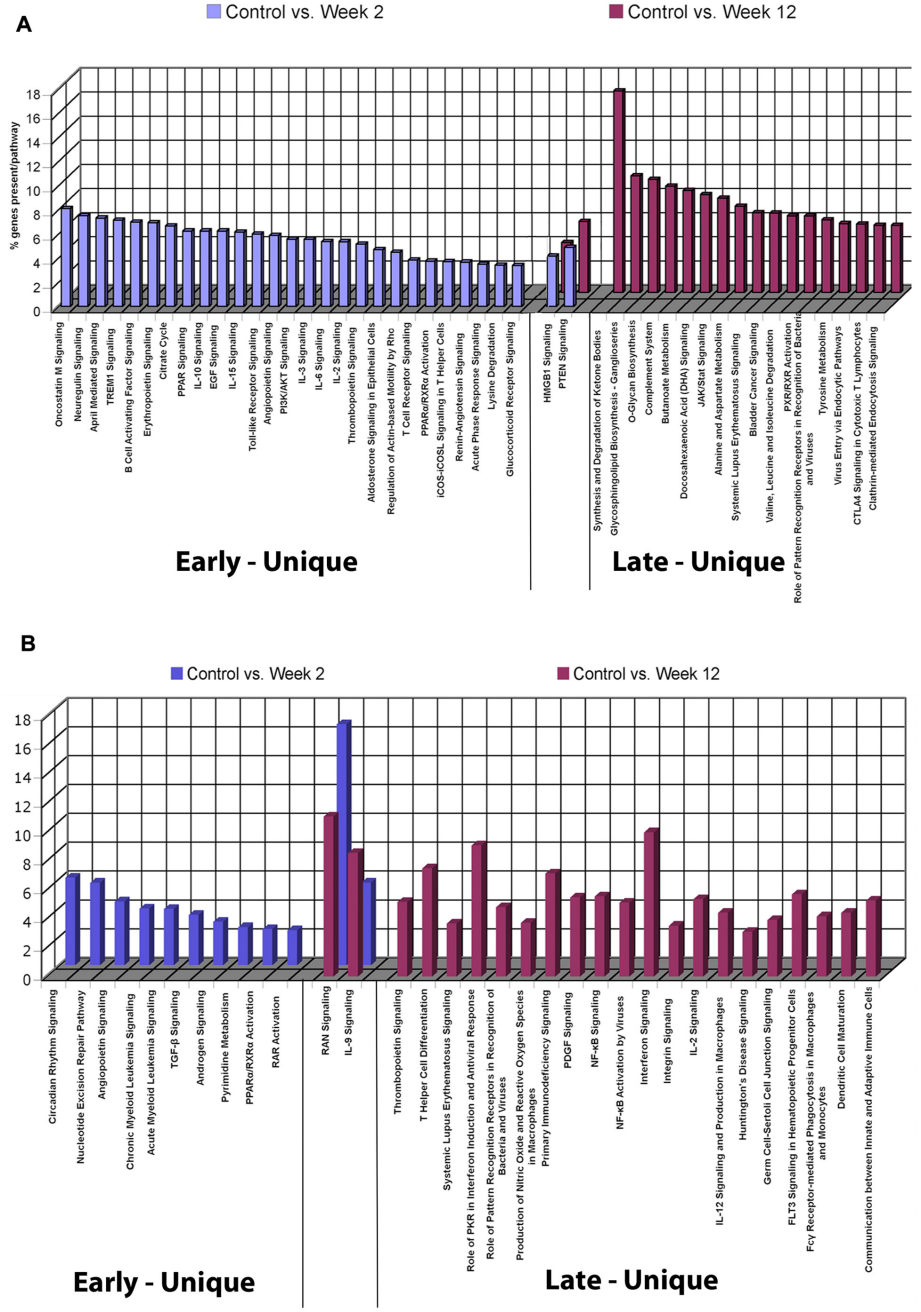
Gene expression profiling and functional analysis of the CD14 monocyte and CD19 B cell subsets. (**A**). Functional pathways populated by genes differentially expressed early (blue), late (red) or shared at both timepoints Post-TX in the CD14 monocyte subset. Y-axis represents % genes present per pathway. (**B**). Functional pathways populated by genes differentially expressed early (blue), late (red) or shared at both timepoints Post-TX in the CD19 B cell subset. Y-axis represents % genes present per pathway.

Mapping of functional pathways demonstrates a distinct contrast with the CD4 and CD8 results. In the CD14 subset, pathways are uniquely populated in the early (27 pathways) or the late phase Post-TX (17 pathways) with only 2 common pathways (PTEN and HMGB1 signaling) (**Figure 6A**). The nature of these CD14 pathways is also distinct between early and late. A large number of immune/inflammatory cytokine signaling and TLR pathways are activated early. Multiple metabolic and synthetic pathways are populated late consistent with the proliferative expansion of the activated CD14 phenotype demonstrated by cytometry.

### CD19 B cells reveal the predominantly late activation of immune signaling pathways

Again, due to low cell yields, CD19 profiling was performed substituting normal donor controls for Pre-TX samples and normalizing. Class comparisons identified 208 significant differentially expressed genes at week 2 (58.1% upregulated) and 389 genes (49.1% upregulated) at week 12 (**Table 6**). Functional mapping populated 32 total pathways (**Figure 6B**) of which only 10 are populated early and 20 are late. Late pathways are rich in immune signaling pathways: interferon, IL2, integrin, PKR-interferon induction, PDGF, TLR receptors, and communications between innate and adaptive immunity. Only two pathways are shared but highly populated: IL9 signaling, a cytokine made by activated T cells, and RAN signaling linked to transport of RNA and proteins from the nucleus.

### Differences in gene expression between whole blood and the purified subsets are due to the impact of neutrophils, eosinophils, and basophils

There is a significant difference in the percent of up/downregulated genes identified by whole blood analysis (11% upregulated; week 2) and the purified CD4, CD8 and CD14 analyses (46%, 52% and 84% upregulated) (**Figure S1 A and B**). The majority of differentially expressed genes in all comparisons are unique to either whole blood or the individual cell subsets underlining the extent of additional information provided by subset analysis in this situation. Secondly, few genes in the subsets move coordinately in opposite directions from whole blood indicating that these values would not cancel out when analyzed in a whole blood sample. As shown in **Figure 1**, cytometry reveals a dramatic depopulation of the major lymphocyte populations immediately following antibody induction therapy. Thus, the explanation for these huge differences in gene expression between whole blood and purified subsets is the impact of the high numbers of neutrophils, eosinophils, and basophils remaining in whole blood.

### Validation of Gene Expression Data By Cytometry-Based Proteomics

Finally, we tested whether we could proteogenomically validate the results of both cytomety and gene expression. We matched 157 different DNA array probesets used for gene expression profiling to the 65 different cell proteins tested by specific antibodies and cytometry. Thus, the novel premise is that the gene expression array data is validated proteomically by the cytometry and the protein expression data identified by cytometry is validated by the gene expression profiling. We found that all the gene expression calls and all the proteins identified were detected by both technologies (**Table S5**).

## Discussion

We have used two complementary strategies to deconvolute the early post-transplant immune response. The first is based on a novel high-throughput laser scanning cytometry technology enabling hundreds of parallel analyses in whole blood [22]. The second approach was purifying constituent blood cell subsets followed by genome-wide gene expression profiling. We studied 10 consecutive kidney transplants with serial blood samples analyzed pre-transplant and at 1, 2, 4, 8 and 12 weeks. Our results provide a unique view of the complex evolution of immune/inflammatory molecular pathways occurring early post-transplant. A critical finding is that the constituent blood cell subsets reveal an entirely new level of detail, effectively deconvoluting a process that is otherwise lost in the mix. A second critical finding is that even in this early period post-TX during the most intense immunosuppression there emerges a clear pattern of immune activation involving memory T cells, monocytes and B cells.

Cytometry results demonstrate the profound reductions in lymphocyte subsets expected with antibody induction. Nonetheless, we demonstrate significant increases in memory CD4 effector cells (CD45RA^−^CD62L^−^) and a reciprocal decrease in naïve CD4, consistent with previous reports [24], [39], [40]. These results support the hypothesis that the protective impact of induction therapy may be limited in this early period by the relative resistance and subsequent expansion of memory effector CD4 cells. In fact, the expansion of memory effector CD4 is supported by the significant upregulation of multiple activation markers. Additionally, induction is less effective at CD8 depletion and these cells also express multiple activation markers and recover more rapidly [14]. The significant reduction in the CD8 terminal effector cell population (CD45RA^+^CD62L^−^) in the context of activation indicates that this early proliferation favors CD8 effector memory and central memory subsets.

A reasonable question is whether cytometry and gene expression data correlate, in effect allowing a proteogenomic validation of both results consistent with our view that these technologies are complementary. Comparison of cytometry and gene expression for multiple pan T cell markers such as CD3e,g,d, TCRab, CD2, CD5, CD6 and CD7 correlated well and similar results were found for pan B cell markers. In fact, gene expression profiling results were validated with all 65 protein antigens measured by cytometry at multiple time points. In turn, all 65 proteins were validated with 157 different probesets detecting the corresponding mRNA transcripts on DNA microarrays. Thus, microarray-based gene expression is representative of the cell proteins expressed and this represents a novel strategy for validating gene expression results by cytometry-based proteomics.

We next investigated gene expression profiles obtained from whole blood. Given the availability of reagents enabling whole blood transcriptome analysis in a clinical setting it was logical to start here. Functional analysis was done using a curated and constantly updated literature database (Ingenuity Pathways Analysis) to populate known pathways with significantly differentially expressed genes. Four classes of functional pathways were populated in the first 12 weeks post-TX: early (1–4 weeks), late (8–12 weeks), consistent (populated at all timepoints) and intermittent (selected timepoints). Most of the pathways were populated either early or consistently and related to primary mechanisms of alloimmunity [24]–[26]. Thus, even in this complicated period, by looking at the evolution of the changes in whole blood gene expression, we identified 2447 genes that change uniquely as a function of time and these mapped to 19 canonical functional pathways (**Figure 3C**). The obvious question is what do we miss in a whole blood analysis because of the dramatic changes in the cell composition that occur after antibody induction in this early period.

The immediate impact of induction is a profound depletion of CD4, CD8 and NK cells, while B cells, eosinophils and neutrophils are suddenly the dominant populations. The ideal way to address this confounding cell admixture challenge is to separate the constituent cell subsets and analyze gene expression from each. We have reviewed in detail the results of these studies. These cell subset-specific results reveal a number of new insights into the mechanisms that may drive the early Post-TX immunity, and are not revealed by whole blood analysis. For example, as shown in **Figure 4B**, we have defined unique early, late and shared pathways for CD4 T cell activation Post-TX. These shared pathways are consistently populated by increasing numbers of differentially expressed genes in the late phase, revealing pathways that are triggered early and then evolve by recruitment and activation of new genes into each pathway with time.

Results for CD8, CD14 and CD19 subsets also demonstrate the evolution of distinct molecular networks as a function of time and cell subset. For example, most of the genes and pathways identified for CD8 are not common to those identified for CD4, underlining the unique nature of the mechanisms driving activation and proliferative expansion of these two T cell subsets. Pathway analysis for CD14 monocytes is consistent with our data showing upregulation of activation markers and published literature that early Post-TX events are dominated by ischemia/reperfusion injury and activation of innate immunity [1], [2], [41]. Thus, the majority of CD14 pathways are activated in the early phase and include signaling via IL10, PPAR, APRIL, ICOS and TLR. In contrast, the majority of CD19 pathways are populated only in the late phase of the early immune response including signaling via: IL9, interferon, IL2, CD4 differentiation, PKR-interferon induction and TLR receptors. Whether these represent coordinated waves of signaling from monocytes to B cells during this early activation of transplant immunity will have to be tested.

This study has several limitations that are important to acknowledge. The data is limited to only 10 patients and 5 healthy donors. To mitigate sample size limitation, patients were enrolled consecutively to avoid selection bias. Also, the serial time sampling of whole blood and purified cell subsets presented here represents over 170 microarrays and thousands of parallel cytometry measurements. Stopping the study at 12 weeks was arbitrary. We also acknowledge that our use of the terms early and late, refer to this 12 week period, which is certainly early in the post-transplant course. The immune response continues to evolve as a function of time and critical events can and will occur later. Future studies, based on the proof of concept provided here, can explore that question productively for the entire first post-transplant year.

There is no attempt to identify specific genes or biomarkers for early transplantation. And the small numbers of patients studied in this pilot preclude any outcome-specific correlations. Rather, we are clear that this study was designed to test the hypothesis that deconvolution of the early Post-TX period is possible by analysis of purified blood cell subsets. First, the results demonstrate that even without subset purification there is considerable information contained within the genome and cytometry data of whole blood samples to identify a number of important candidate pathways. Second, subset-based deconvolution allows a novel correlation of the profound changes in blood cell composition created by antibody induction with the molecular pathways activated in critical immune cell subsets. These subset-specific changes illuminate a very complex, early phase of immunity that includes activation and proliferative expansion of the memory effector and regulatory cells that determine the phenotype and outcome of the kidney transplant. In fact, it is striking that in this first 12 weeks under the most intense levels of immunosuppression achieved at any time post-transplant that there is such a dramatic activation of CD4, CD8, CD14 and B cells and particularly of the memory effector subsets. With these results, it is now justifiable to consider a larger investment in serial sampling and blood cell subset analysis of kidney transplant patients that should extend its reach to at least 12 months post-transplantation. Ultimately, the question for the field is whether these networks of immune activation represent the necessary repopulation of the immune system in response to early antibody-mediated destruction, and if so, what are the implications of this activation for the evolution and regulation of the post-transplant immune response? The current clinical reality is that despite significant reductions in the incidence of acute rejection over the last two decades, we still have not found a protocol that results in tolerance with drug withdrawal or even indefinite long term graft survival on immunosuppression.

## Materials and Methods

### Ethics Statement

All the studies in this manuscript were covered by Human Subjects Research Protocols approved by the Center's Institutional Review Board and by the IRB of The Scripps Research Institute. Informed written consent was obtained from all study subjects in the study.

### Patient population and treatment regiment

The immunosuppressive protocol was: methylprednisolone (60 mg/days 1–4); rabbit polyclonal anti-thymocyte globulin (Thymoglobulin®; 6 mg/kg in 3 doses); mycophenolate mofetil (CellCept®); and tacrolimus (Prograf®). Peripheral blood from 10 transplant patients was collected with IRB approved informed consent at the Scripps Center for Organ and Cell Transplantation immediately prior to administration of immunosuppression and transplantation and at weeks 1, 2, 4, 8, and 12. Whole Blood was collected into PAXgene (PreAnalytix, Franklin Lakes, NJ) or EDTA-coated tubes for separation into cell subsets using positive cell selection with magnetic Dynal beads (Invitrogen, Carlsbad, CA). Blood Samples from 5 healthy control subjects from our Normal Blood Drawing Service represented by 2 males and 3 females, 25–45 years of age, were collected following the same protocol at a single time point.

### Cytometry

Cytometry analysis was performed on the SurroScan™ system (SurroMed/PPD Biomarker Discovery Sciences) comprised of 49 three-color cell surface assays performed by microvolume laser scanning cytometry as described previously [21]–[23], [42]. Template gates were established using FlowJo™ software (Tree Star, Inc., Ashland, OR) customized for PPD. Gating information was applied for each assay using CytoSuite™ analysis software to generate cell counts and antigen intensity data. **Table S6** provides a summary of target antigens used in this study for each major cell population**.** Template gates were established with healthy controls (Stanford Blood Bank) and confirmed to work with study samples (**Figure S2**). Control experiments demonstrated that residual thymoglobulin did not interfere with the cytometry measurements (not shown). We evaluated 1083 statistical variables from cell counts and cell surface antigen intensities. Within group comparisons were performed as paired two-group comparisons to identify differences associated with transplantation and immunosuppression independent of outcome. If all variables were independent, 11 would be expected to be different by chance at a univariate p-value of <0.01.

### Gene expression profiling and functional mapping

RNA was extracted and hybridized to Affymetrix U133 Plus 2.0 GeneChips (Affymetrix, Santa Clara, CA). Normalized signals were used for class comparisons of variance by two-way t-tests for two sample comparisons and parametric univariate F-tests for multiple sample comparisons (p<0.001, FDRs<2%; BRB-ArrayTools, (http://linus.nci.nih.gov/BRB-ArrayTools.html) to identify significantly differentially expressed genes. All tests done were based on comparing the differences in normalized, mean log-transformed intensities between classes of samples. Heatmaps were generated using Cluster and Treeview [43]. Functional mapping was performed using Ingenuity Pathway Analysis (IPA, Ingenuity® Systems, Redwood City, CA, http://www.ingenuity.com). All gene expression files are available at the NIH GEO site, submission number GSE24223. For detailed description of cytometry, statistical methods and gene analysis refer to **Methods S1.**

## Supporting Information

Figure S1 Comparisons of gene expression between 3 cell subsets (CD4, CD8 and CD14) and whole blood. (A) The Venn diagram represents the gene expression overlaps between whole blood (WB) and the purified CD4, CD8, and CD14 subsets, showing genes downregulated early. The number in parenthesis next to each cell subset represents the total number of downregulated genes at that timepoint Post-TX. The data shows that the majority of genes differentially expressed early in each cell type is unique to that cell type. (B) The Venn diagram represents the gene expression overlaps between whole blood (WB) and the purified CD4, CD8, and CD14 subsets, showing genes downregulated late in the 12 week period post transplantation. The number in parenthesis next to cell subset represents the total number of downregulated genes at that timepoint Post-TX. The data shows that the majority of genes differentially expressed late in each cell type is unique to that cell type.
(1.08 MB TIF)

Figure S2 Representative laser scanning cytometry plots with standard gates for the major cell populations. (A) Total T cells identified with CD3; (B) CD4 and CD8 T cells after gating on CD3; (C) NK cells identified as CD3 negative (not shown) and CD2 and CD56 positive; (D) B cells (CD20) and monocytes (CD14); (E) Granulocytes (CD16) and monocytes (CD14); and, (F) granulocytes (CD16) and eosinophils, identified as CD16 negative and CD66b positive. All of these examples are from whole blood staining with healthy controls. When a population was measured in more than one assay, averages of the absolute cell counts were used for the comparative statistics.
(2.22 MB TIF)

Table S1 Whole Blood multivariate gene expression
(1.62 MB XLS)

Table S2 134 multivariate whole blood genes that populate 19 significant functional pathways
(0.12 MB XLS)

Table S3 Significant pathways (p<0.05) identified in Ingenuity Pathway Analysis populated by genes from whole blood as a function of the serial time points sampled
(0.05 MB XLS)

Table S4 Comparison analysis of functional pathways populated either early, late or at both stages Post-Tx in CD8 subset
(0.02 MB XLS)

Table S5 Cytometry Antigen Gene Expression
(0.14 MB XLS)

Table S6 Target Antigens for Cellular Assays.
(0.03 MB DOC)

Methods S1 Supplemental methods
(0.03 MB DOC)
