# Supplementary figures and images for: Deconvoluting Post-Transplant Immunity: Cell Subset-Specific Mapping Reveals Pathways for Activation and Expansion of Memory T, Monocytes and B Cells
Source: PLoS One. 2010 Oct 14;5(10):e13358. doi: 10.1371/journal.pone.0013358 (PMC2954794; doi:10.1371/journal.pone.0013358)

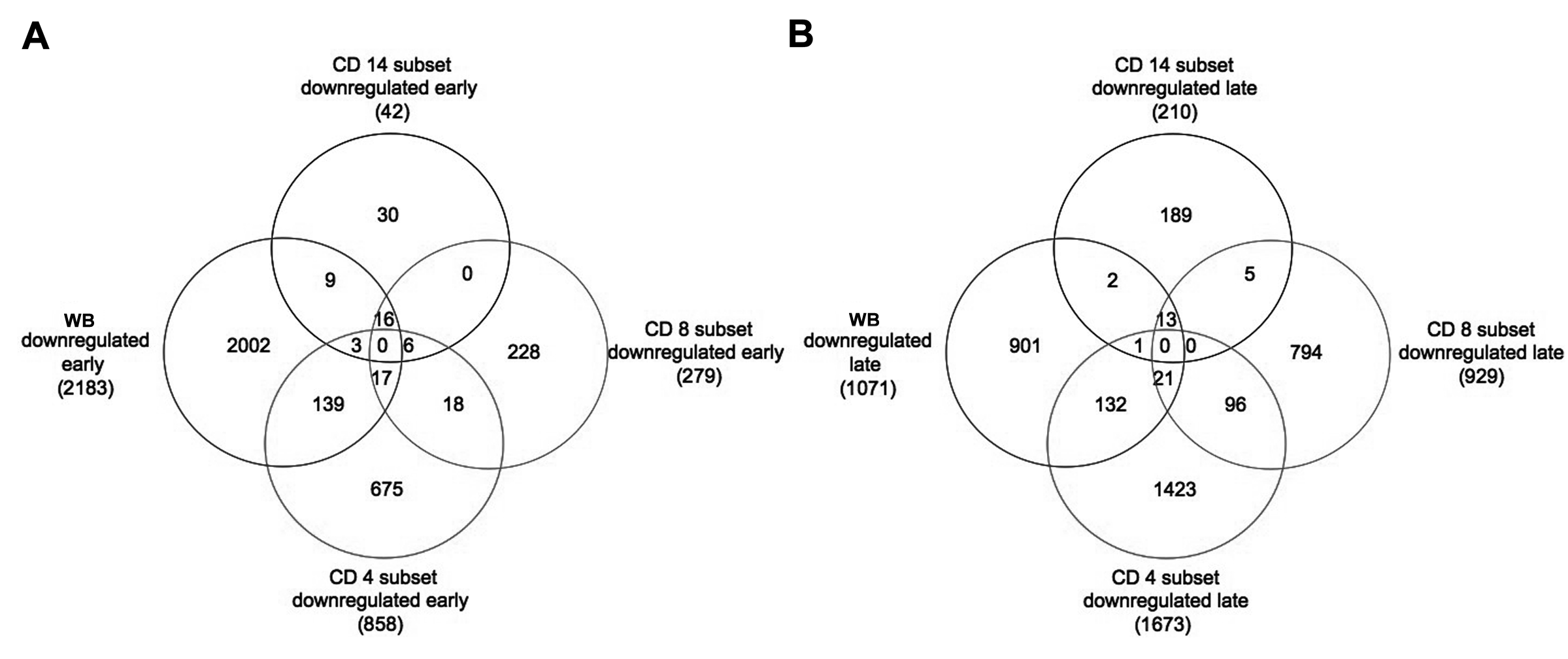

Supplement: Figure S1 — Comparisons of gene expression between 3 cell subsets (CD4, CD8 and CD14) and whole blood. (A) The Venn diagram represents the gene expression overlaps between whole blood (WB) and the purified CD4, CD8, and CD14 subsets, showing genes downregulated early. The number in parenthesis next to each cell subset represents the total number of downregulated genes at that timepoint Post-TX. The data shows that the majority of genes differentially expressed early in each cell type is unique to that cell type. (B) The Venn diagram represents the gene expression overlaps between whole blood (WB) and the purified CD4, CD8, and CD14 subsets, showing genes downregulated late in the 12 week period post transplantation. The number in parenthesis next to cell subset represents the total number of downregulated genes at that timepoint Post-TX. The data shows that the majority of genes differentially expressed late in each cell type is unique to that cell type. (1.08 MB TIF) [file pone.0013358.s001.tif]

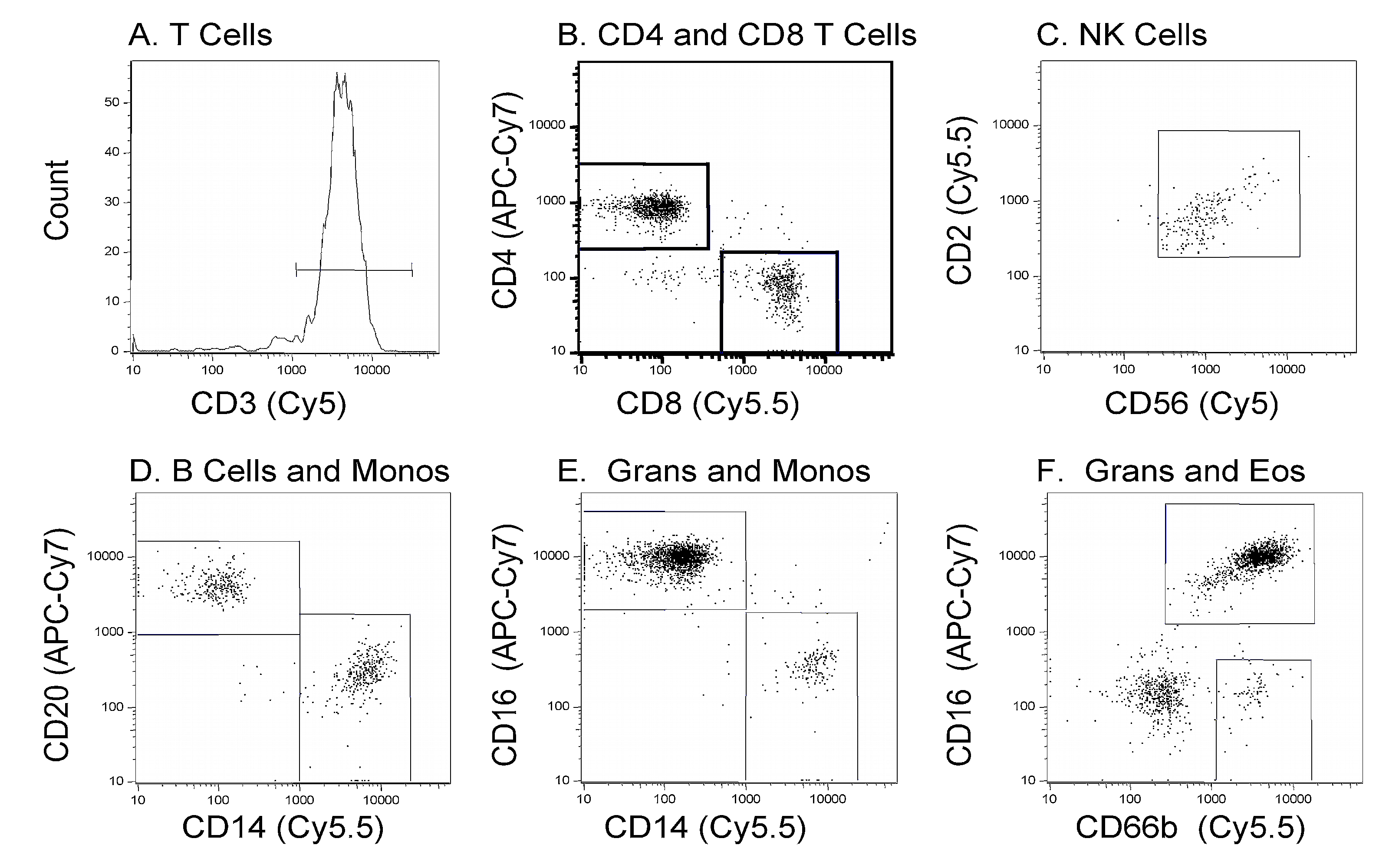

Supplement: Figure S2 — Representative laser scanning cytometry plots with standard gates for the major cell populations. (A) Total T cells identified with CD3; (B) CD4 and CD8 T cells after gating on CD3; (C) NK cells identified as CD3 negative (not shown) and CD2 and CD56 positive; (D) B cells (CD20) and monocytes (CD14); (E) Granulocytes (CD16) and monocytes (CD14); and, (F) granulocytes (CD16) and eosinophils, identified as CD16 negative and CD66b positive. All of these examples are from whole blood staining with healthy controls. When a population was measured in more than one assay, averages of the absolute cell counts were used for the comparative statistics. (2.22 MB TIF) [file pone.0013358.s002.tif]
