# Supplementary material for: Deconvoluting Post-Transplant Immunity: Cell Subset-Specific Mapping Reveals Pathways for Activation and Expansion of Memory T, Monocytes and B Cells
Source: PLoS One. 2010 Oct 14;5(10):e13358. doi: 10.1371/journal.pone.0013358 (PMC2954794; doi:10.1371/journal.pone.0013358)
Supplement: Table S6 — Target Antigens for Cellular Assays. (0.03 MB DOC) [file pone.0013358.s008.doc]

#### Supplementary Table 6. Target Antigens for Cellular Assays.

| **Major Cells** | **Major Marker** | **Subsetting Antigen** |
| --- | --- | --- |
| T Cells | All: CD2, CD3, CD5  Helper: CD4 T  Cytotoxic: CD8 T | CCR4, CCR5, CD6, CD25, CD27, CD28, CD38, CD44 CD45RA, CD45RO, CD49d, CD56, CD57, CD60, CD62L, CD69, CD86, CD95, CD127, CD132, CD134, CD154, CD183, CD197, HLA-DR, IL-15Ra, TCR αβ, TCRγδ, |
| **B Cells** | CD19, CD20 | CD5, CD38, CD40, CD44, CD95, HLA-DP, DQ, DR and PAN |
| **NK Cells** | CD56, NKB1 | CD2, CD94, CD122, CD161 |
| Granulocytes **Eosinophils** | CD16,  CD66b | CD45, CD64, CD52, CD66b, CD132 |
| **Monocytes** | CD14 | CD4, CD25, CD31, CD38, CD40, CD44, CD45, CD64, CD69, CD86, CD282, CD284, HLA-DP, DQ, DR and PAN |
| **Platelets** | CD41a | CD62P, CD31 |
