# Supplementary material for: Deconvoluting Post-Transplant Immunity: Cell Subset-Specific Mapping Reveals Pathways for Activation and Expansion of Memory T, Monocytes and B Cells
Source: PLoS One. 2010 Oct 14;5(10):e13358. doi: 10.1371/journal.pone.0013358 (PMC2954794; doi:10.1371/journal.pone.0013358)
Supplement: Methods S1 — Supplemental methods (0.03 MB DOC) [file pone.0013358.s009.doc]

**Detailed Supplemental Methods**

**Cytometry**

Cytometry analysis was performed in collaboration with Dr. Aaron Kantor at Biomarker Discovery Sciences Division of PPD (Menlo Park, CA) on SurroScan™ system (SurroMed/PPD Biomarker). The cellular assay protocol included 49 three-color cell surface assays performed by microvolume laser scanning cytometry (MLSC) on SurroScan™ system. Three different fluorophores, Cy5, Cy5.5 and the tandem dye Cy7-APC were coupled to individual monoclonal antibodies specific for different cellular antigens in each assay. Each fluorophore was measured in a separate detection channel. The antibody-dye reagents were combined into pre-made cocktails and all assays were homogeneous, without removal of unreacted antibody reagents. Aliquots of either whole or red blood cell-lysed blood from EDTA collection tubes were added to 96-well micro-titer plates containing the appropriate reagent cocktails, incubated in the dark at room temperature for 20 minutes, diluted with an appropriate buffer and loaded into Flex32™ capillary arrays (SurroMed/PPD Biomarker) and analyzed with SurroScan™. Images were converted to a list-mode data format with in-house software. Fluorescence intensities were compensated for spectral overlap of the dyes so values were proportional to antigen density.

As part of the development of Surroscan for this specific study, the quality and baseline measures for the 49 different three-color cellular assays we chose to use in this study were established with ten normal donors obtained from the blood bank (data not shown). These panels also included 15 reagent controls. Assays were qualified for next day staining and scanning including testing of the impact of shipping on expression of the markers. All samples were prepared and shipped in the same packaging that had been optimized for this purpose by PPD for another clinical study. Based on this work, standard template gates were established for the Surroscan instrument using these results plus additional staining controls for all individual reagents and underlying two-color combinations. Template gates were established using FlowJo™ cytometry analysis software (Tree Star, Inc., Ashland, OR) customized for PPD Biomarker/SurroMed to enable uploading of gates to their Oracle database. Gating information was stored in the database and applied to the scan data for each assay using CytoSuiteTM database-driven cytometry analysis software in order to generate the resulting cell count and antigen intensity data. A summary of the target antigens for each of the major cell populations is provided in **Supplemental Table 6.**

**Cytometry variables and statistical analysis.**

Our evaluation included 1083 variables from cell counts and cell surface antigen intensities. Multiple measures of the same cell population (e.g., CD4 T cells) were combined into a single average for the analysis. These results from the 49 cell surface assays were used in the statistical analysis. Among the assays, 0.53% was marked as invalid due to technical difficulties and was excluded from the statistical analysis. An additional 3.45% required non-standard gates. Cell counts from non-standard gates were generally not affected, but cell surface expression results may have a larger variation. Within group comparisons for transplantation subjects were performed as paired two-group comparisons designed to identify differences associated with transplantation and immunosuppression independent of outcome.

**Gene expression profiling and functional analysis**

RNA was extracted from PAXgene tubes using the PAXgene Blood RNA system (PreAnalytix) and GlobinClear (Ambion) or Trizol (Invitrogen) from separated cell subsets. Biotinylated cRNA was prepared with Ambion MessageAmp Biotin II kit (Ambion) and hybridized to Affymetrix Human Genome U133 Plus 2.0 GeneChips (Affymetrix, Santa Clara, CA). Data was collected as CEL files. Normalized signals that were generated using a quantile normalization strategy with Robust Multichip Average (RMA) were used for class comparisons of variance in BRB-ArrayTools (http://linus.nci.nih.gov/BRB-ArrayTools.html). These comparisons are statistically tested by a univariate t-test if between two data sets and a parametric univariate F-test if involving multiple. In both cases, p values are set at <0.001, FDRs <2% to identify significantly differentially expressed genes. The output of the analysis was a list of annotated significant genes. Heatmaps were generated using Cluster and Treaview. Functional analyses were performed using Ingenuity Pathway Analysis (IPA, Ingenuity® Systems, Redwood City, CA; http://www.ingenuity.com), a constantly curated database of published literature on gene functions and interactions. Genes identified by BRBArrayTools as significantly differentially expressed were uploaded for each time point analysis into IPA to populate functional pathways. Only significant functional pathways (p <0.05) were considered for analysis. The significance provided statistical confidence that the pathways are associated with uploaded genes. Percentage of genes present per pathway was calculated from the ratio of the number of molecules in a given pathway that meet cutoff criteria, divided by total number of molecules that make up the pathway. Percentage of genes provides the amount of association while significance gives confidence of association. All gene expression files are available at the NIH GEO site.
